# Assessing red blood cell distribution width in Vietnamese heart failure patients: A cross-sectional study

**DOI:** 10.1371/journal.pone.0301319

**Published:** 2024-07-23

**Authors:** Hai Nguyen Ngoc Dang, Thang Viet Luong, Mai Thi Thu Cao, Vinh Trung Bui, Thanh Thien Tran, Hung Minh Nguyen

**Affiliations:** 1 The Faculty of Medicine, Duy Tan University, Da Nang, Vietnam; 2 Cardiovascular Center, Hue Central Hospital, Hue, Vietnam; 3 University of Medicine and Pharmacy, Hue University, Hue, Vietnam; 4 Vietnam National Heart Institute, Bach Mai Hospital, Hanoi, Vietnam; University of Montenegro-Faculty of Medicine, MONTENEGRO

## Abstract

**Background:**

Heart failure (HF) is becoming a growing public health concern. Diagnostic tests for determining the severity of HF often come with high costs and require specialized expertise, which makes it difficult to assess HF severity, especially in low-income countries or at primary healthcare facilities. Recently, red blood cell distribution width (RDW) has emerged as a promising, easily accessible marker associated with HF severity. The study aimed to assess changes in RDW levels in HF patients and the diagnostic value of RDW in detecting acute heart failure (AHF) among HF patients.

**Methods:**

We conducted a cross-sectional examination involving 351 participants divided into HF and non-HF cohorts. HF was defined and categorized according to the diagnostic and treatment guidelines for AHF and chronic heart failure (CHF) set forth by the European Society of Cardiology (2021). Univariate and multivariate analysis of factors associated with AHF was performed.

**Results:**

The study revealed that HF patients displayed higher median RDW levels (14.90% [13.70–17.00]) compared to non-HF individuals (13.00% [12.23–13.78]). RDW was notably elevated in HF patients with left ventricular ejection fraction < 50% compared to those with left ventricular ejection fraction ≥ 50%. ROC curve analysis of RDW for AHF detection identified a cutoff value of 13.85%, with a sensitivity of 86.05% and specificity of 47.18%, statistically significant at p < 0.001. RDW > 13.85% was identified as an independent risk factor for AHF in patients with HF, with odds ratios of 2.644 (95% CI, 1.190–5.875; p = 0.017).

**Conclusion:**

The study revealed significant RDW variations in patients with CHF and AHF compared to the control group. These findings suggest that RDW could be a biomarker for detecting HF severity.

## Introduction

Heart failure (HF) is a complex and life-threatening syndrome characterized by significant morbidity and mortality, limited functional capacity and quality of life, and substantial economic burden [1]. Additionally, HF has emerged as an increasingly critical public health concern. Given the aging population and enhanced survival rates following acute myocardial infarction, these trends are expected to persist [2]. HF has been recognized as a global pandemic, affecting an estimated 64.3 million individuals worldwide in 2017 [3]. Despite therapeutic advancements, HF remains a primary contributor to morbidity and mortality on a global scale [4].

The early detection and accurate diagnosis of HF are crucial for optimizing the treatment and prognosis of patients with this condition. Currently, HF diagnosis relies primarily on echocardiography and patients’ presenting symptoms, yet a definitive prognostic indicator for mortality among HF patients is lacking [5]. Biomarkers have shown promise in enhancing predictive capabilities alongside clinical evaluation in chronic heart failure (CHF) and acute heart failure (AHF) patients, such as B-type natriuretic peptides or troponins [6–8]. However, in Vietnam, the costs associated with these tests are prohibitive, and they are available only in large medical centers, presenting a significant challenge to their widespread application.

Red blood cell distribution width (RDW) is a parameter that reflects the variability in red blood cell size (anisocytosis). It is easily obtained from a complete blood count and represents a simple, cost-effective measure [9]. RDW is increasingly recognized as a marker for predicting the onset and progression of HF. In HF patients, anisocytosis may signify a homeostatic response to the disease, potentially indicating a link between ineffective erythropoiesis and chronic inflammation [10]. Traditionally, RDW has been underappreciated and primarily used to differentiate specific causes of anemia [11]. However, recent clinical evidence suggests that changes in RDW are associated with the development and adverse outcomes of stroke and cardiovascular disease [9,10,12,13]. RDW has been correlated with hospitalization rates and poor prognosis, particularly in HF patients [14,15]. RDW is a readily applicable blood parameter for primary healthcare facilities, especially in Vietnam. Nonetheless, most existing evidence originates from regions such as the United States, Europe, Japan, and China, excluding Vietnam [5,16,17].

We hypothesize that RDW, which can be easily obtained from a complete blood count as a simple and cost-effective measure, may undergo changes in HF patients and its value in predicting their severity. Therefore, this study aimed to investigate RDW levels in hospitalized Vietnamese HF patients and assess the ability of RDW to predict the severity of HF in this population.

## Materials and methods

### Study population

**Fig 1:**
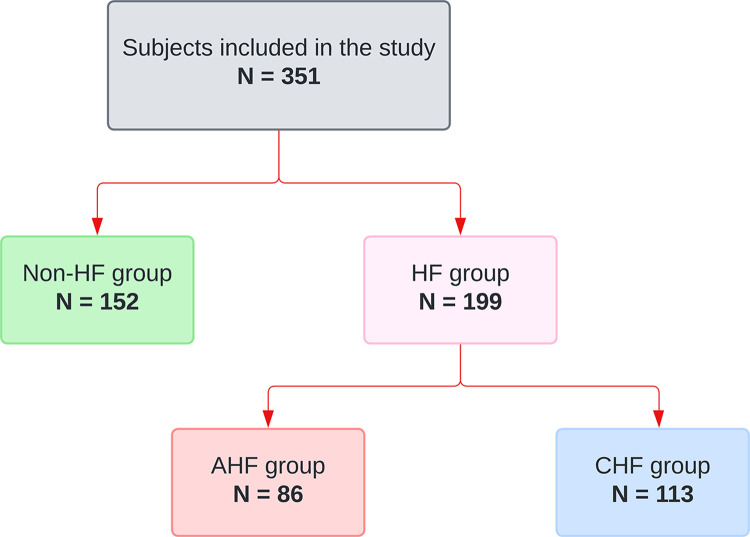
The research flowchart divides the research subjects into subgroups according to group. HF: Heart failure; CHF: Chronic heart failure; AHF: Acute heart failure.

A cross-sectional study was conducted at Hue Central Hospital from 27/02/2022 to 27/02/2024, involving 351 patients categorized into two groups: those with HF and those without HF (**Fig 1**). CHF and AHF were defined and classified based on the criteria outlined in the European Society of Cardiology guidelines for diagnosing and treating AHF and CHF [18]. The control group consisted of patients with no history or symptoms of HF, left ventricular ejection fraction (LVEF) ≥ 50%, and N-terminal pro B-type natriuretic peptide (NT-proBNP) < 125 pg/mL. Additionally, patients who had received blood transfusions or iron supplementation during hospitalization and those with a positive osmotic fragility test were excluded from the study. The project received approval from Hue Central Hospital and the Institutional Review Board of Duy Tan University (No: Đ23-24Y3-2) and adhered to the principles of the Declaration of Helsinki (2013 version).

### Data collection

The baseline evaluation of the patients included demographic characteristics and clinical assessments, including medical history (hypertension, diabetes mellitus, dyslipidemia, coronary artery disease, chronic kidney disease, valvular heart disease, liver disease, and cardiac arrhythmias, as well as medication usage). Additionally, other clinical data, such as body mass index (BMI), waist-hip ratio (WHR), heart rate, systolic blood pressure (SBP), diastolic blood pressure (DBP), and New York Heart Association (NYHA) classification, were collected. These details were routinely collected upon admission and hospitalization utilizing a pre-established registry questionnaire.

The echocardiographic procedure adhered to the guidelines outlined by the American Society of Echocardiography for conducting a comprehensive transthoracic echocardiographic examination in adults performed by expert echocardiographers [19].

Laboratory analyses were performed on fasting venous blood specimens and included NT-proBNP, red blood cell (RBC), hematocrit (HCT), hemoglobin (Hb), mean cell volume (MCV), mean corpuscular hemoglobin (MCH), mean cell hemoglobin concentration (MCHC), RDW, platelet count (PLT), alanine aminotransferase (ALT), aspartate transaminase (AST), glucose, urea, high-sensitive cardiac troponin T (hs-cTnT), hemoglobin A1c (HbA1C), total cholesterol, triglyceride, low-density lipoprotein cholesterol (LDL-C), high-density lipoprotein cholesterol (HDL-C), non-high-density lipoprotein cholesterol (non-HDL-C), potassium level, sodium level, chloride level, and estimated glomerular filtration rate using CKD-EPI 2021 (eGFR, mL/min per 1.73 m^2^) [20].

### RDW measurement

**Fig 2:**
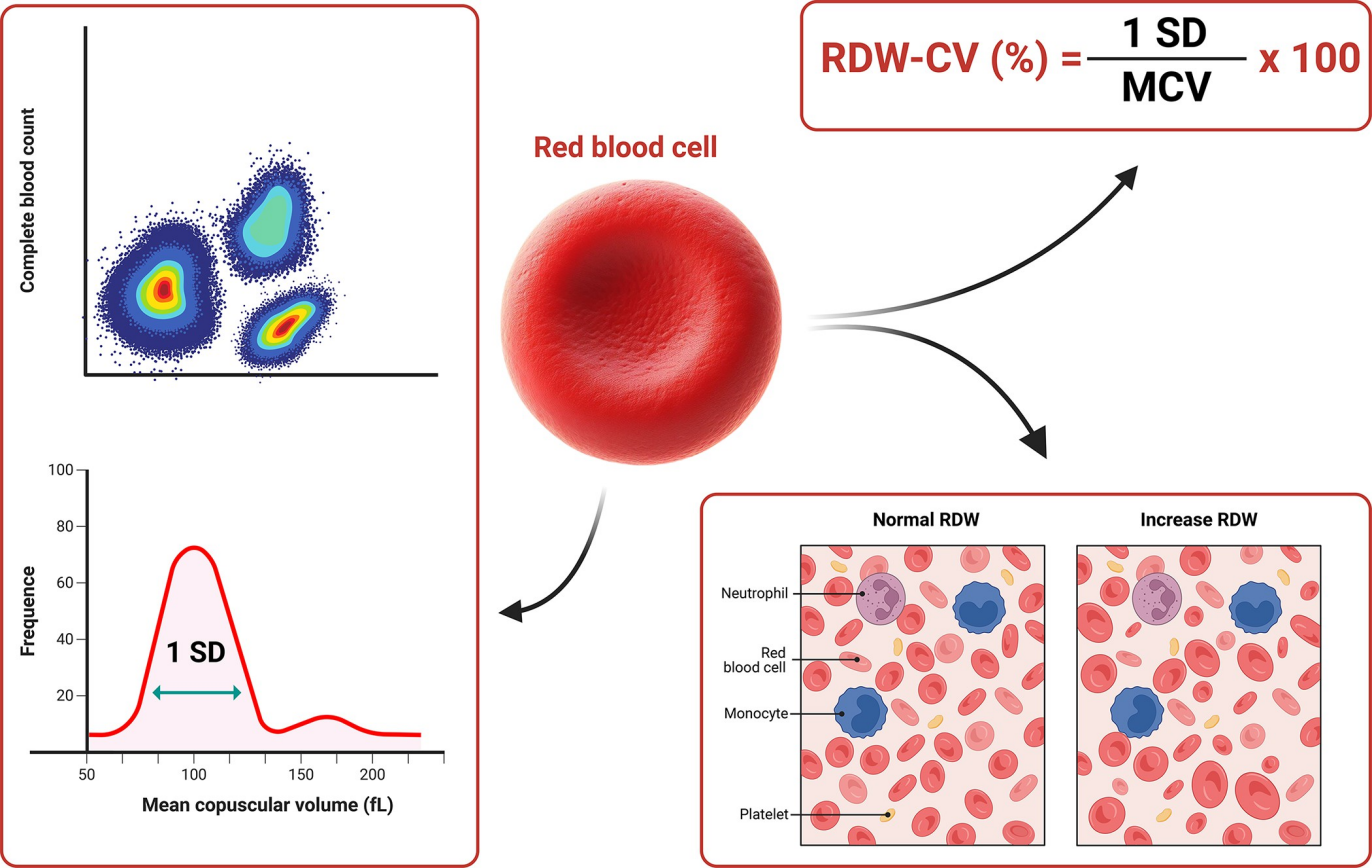
The RDW was calculated by the RDW-CV formula, with 1 SD divided by the MCV. RDW-CV: Red blood cell distribution width coefficient of variation; SD: Standard deviation; MCV: Mean corpuscular volume.

Blood samples were collected from 2 mL of the peripheral vein using an ethylene diamine tetraacetic acid tube within 6 hours after admission and analyzed using a Sysmex XS-1000i automated hematology analyzer (Japan) located in the Hematology Department of Hue Central Hospital. The RDW index was derived from the complete blood count obtained upon patient admission to the hospital. Two RDW parameters were calculated to measure the extent of anisocytosis: the standard deviation (SD) and the coefficient of variation (CV). The RDW-CV has been widely investigated and calculated using the following formula: RDW-CV = (SD of erythrocyte volume/MCV) × 100 [15]. The standard reference range for RDW in the hospital laboratories that participated in the study was 11.6–14.8%. In this study, we reported the RDW-CV and used the RDW to represent it. Detailed illustration of RDW calculation through complete blood count and the abnormalities of the RDW index are depicted in **Fig 2**.

### Statistical analysis

All the statistical analyses were performed using SPSS Version 26 (IBM, New York, United States), MedCalc Software Version 22.019 (MedCalc Software, Ostend, Belgium), and GraphPad Prism Version 10 (GraphPad Software, Boston, United States). We assessed data normality using the Kolmogorov‒Smirnov test. Normally distributed continuous variables are presented as the mean ± SD, while nonnormally distributed variables are described as median values with interquartile ranges. Categorical variables are reported as frequencies and percentages. Fisher’s exact test was used to assess intergroup differences in categorical variables. In contrast, differences in continuous variables were analyzed using either the Student’s t-test or the Mann‒Whitney U test, as appropriate. Missing data were excluded from the analyses. Spearman’s correlation coefficient (r_s_) and its corresponding p value were calculated to explore the correlation between continuous variables. The area under the curve (AUC) was determined using the Wilson/Brown method to detect AHF in HF patients. AUC comparison was conducted to evaluate the diagnostic performance of RDW compared to other biochemical tests, employing the Hanley and McNeil method [21]. Univariate logistic regression was used to calculate odds ratios to determine the strength and direction of the association between complete blood count indices and AHF in HF patients. Clinical confounding factors and significant variables (p < 0.05) in the univariate analysis were included in the multivariable model to evaluate the independent effects of RDW on AHF in patients with CHF, and a multivariate logistic regression analysis was performed. All the statistical tests were two-sided, with a significance level of < 0.05.

## Results

### Baseline characteristics

A study involving 351 subjects revealed significant differences in complete blood count, such as RBC, Hb, and RDW-CV, between HF and non-HF patients. Detailed information regarding these indices is presented in **Tables 1** and **2**.

**Table 1:** Baseline characteristics of the study groups.

Characteristics	Non-HF (n = 152)	HF (n = 199)	p value
**Baseline demographic and clinical features**			
**Age (Years)**	66.6 ± 12.1	70.1 ± 14.1	0.008
**Female**	115 (75.7)	119 (59.8)	0.002
**Heart rate (bpm)**	82 [76–90]	81 [75–95]	0.838
**SBP (mmHg)**	140 [130–170]	130 [110–150]	< 0.001
**DBP (mmHg)**	80 [80–90]	80 [70–80]	< 0.001
**BMI (kg/m** ^**2**^ **)**	22.4 [20.2–25.3]	21.0 [19.2–23.1]	< 0.001
**WHR**	1.0 [0.9–1.0]	1.0 [0.9–1.0]	0.681
**NYHA**	NA	2 [2–3]	NA
**LVEF (%)**	62.5 [61.0–64.3]	55.0 [36.0–61.0]	< 0.001
**Smoking**	27.0 (17.8)	31.0 (15.6)	0.664
**Alcohol**	12.0 (7.9)	16.0 (8.0)	0.063
**Diabetes**	20.0 (13.2)	51.0 (25.6)	0.005
**Dyslipidemia**	39.0 (25.7)	66.0 (33.2)	0.158
**Hypertension**	116.0 (76.3)	130 (65.3)	0.034
**Stent**	3.0 (2.0)	47.0 (23.6)	< 0.001
**CABG**	0.0 (0.0)	11.0 (5.5)	0.003
**Atrial fibrillation**	1.0 (0.7)	39.0 (19.6)	< 0.001
**Stroke**	8.0 (5.3)	15.0 (7.5)	0.515
**CKD**	3.0 (2.0)	21.0 (10.6)	0.001
**Medication usage**			
**Antiplatelet**	17.0 (11.2)	25.0 (12.6)	0.005
**Beta blockers**	24.0 (15.8)	44.0 (22.1)	< 0.001
**ACEi/ARB**	81.0 (53.3)	59.0 (29.7)	0.299
**MRA**	4.0 (2.6)	42.0 (21.1)	< 0.001
**ARNI**	0.0 (0.0)	7.0 (3.5)	0.001
**SGLT2i**	4.0 (2.6)	26.0 (13.1)	< 0.001
**Digoxin**	0.0 (0.0)	7.0 (3.5)	0.001

Values are presented as the mean ± standard deviation, median [25th interquartile - 75th interquartile], or number (%) as appropriate. SBP: Systolic blood pressure; DBP: Diastolic blood pressure; BMI: Body mass index; WHR: Waist–hip ratio; NYHA: New York Heart Association; LVEF: Left ventricular ejection fraction; CABG: Coronary artery bypass graft; CKD: Chronic kidney disease; ACEi/ARB: Angiotensin-converting enzyme inhibitor/angiotensin receptor blocker; MRA: Mineralocorticoid receptor antagonist; ARNI: Angiotensin receptor neprilysin inhibitor; SGLT2i: Sodium-glucose cotransporter 2 inhibitor.

**Table 2:** Laboratory tests of the study groups.

Laboratory parameters	Non-HF (n = 152)	HF (n = 199)	p value
**WBC (K/μL)**	7.8 [6.5–9.6]	7.6 [6.0–9.9]	0.742
**RBC (M/μL)**	4.5 [4.2–4.7]	3.8 [3.3–4.3]	< 0.001
**Hb (g/dL)**	13.1 [12.1–14.0]	10.9 [8.7–12.5]	< 0.001
**HCT (%)**	39.5 [37.5–41.5]	33.5 [27.3–38.2]	< 0.001
**MCV (fL)**	89.4 [86.1–92.6]	88.3 [81.1–92.8]	0.088
**MCH (pg)**	29.9 [28.8–30.9]	29.0 [26.1–30.6]	0.002
**MCHC (g/dL)**	33.2 [32.5–34.1]	32.6 [31.7–33.4]	< 0.001
**RDW-CV (%)**	13.00 [12.23–13.78]	14.90 [13.70–17.00]	< 0.001
**PLT (K/μL)**	251 [215–294]	230 [184–287]	0.006
**MPV (fL)**	9.4 [8.3–9.9]	8.7 [8.0–9.5]	0.001
**Glucose (mmol/L)**	6.3 [5.3–7.8]	6.4 [5.3–7.7]	0.614
**HbA1c (%)**	6.3 [5.6–8.2]	6.5 [5.8–8.1]	0.634
**AST (U/L)**	24.0 [19.3–31.4]	29.7 [21.5–41.2]	0.002
**ALT (U/L)**	22.5 [15.4–33.2]	22.0 [14.1–31.8]	0.567
**Cholesterol (mmol/L)**	5.1 ± 1.1	4.4 ± 1.1	< 0.001
**Triglyceride (mmol/L)**	1.5 [1.0–2.4]	1.2 [0.9–1.8]	< 0.001
**HDL-C (mmol/L)**	1.3 ± 0.3	1.2 ± 0.3	0.013
**non-HDL-C (mmol/L)**	3.8 ± 1.0	3.2 ± 1.0	< 0.001
**LDL-C (mmol/L)**	3.2 [2.5–3.8]	0.9 [0.6–2.4]	< 0.001
**Urea (mmol/L)**	4.7 [3.7–6.2]	6.3 [5.0–8.6]	< 0.001
**Creatinine (μmol/L)**	67 [55–79]	84 [61–120]	< 0.001
**eGFR (mL/min/1.73m** ^**2**^ **)**	86 [71–95]	67 [46–88]	< 0.001
**Potassium (mmol/L)**	3.4 [3.2–3.8]	3.7 [3.3–4.2]	< 0.001
**Sodium (mmol/L)**	138 [134–139]	136 [134–140]	0.278
**Chloride (mmol/L)**	101 [99–103]	101 [97–105]	0.478
**hs-cTnT (ng/L)**	7.0 [6.0–11.0]	18.5 [9.0–31.5]	< 0.001
**NT-proBNP (pg/mL)**	64 [32–99]	1807 [487–8353]	< 0.001

Values are presented as the mean ± standard deviation, median [25th interquartile - 75th interquartile] as appropriate. WBC: White blood cell; RBC: Red blood cell; Hb: Hemoglobin; HCT: Hematocrit; MCV: Mean corpuscular volume; MCH: Mean corpuscular hemoglobin; MCHC: Mean corpuscular hemoglobin concentration; RDW-CV: Red blood cell distribution width coefficient of variation; PLT: Platelet; MPV: Mean platelet volume; HbA1c: Hemoglobin A1c; AST: Aspartate aminotransferase; ALT: Alanine aminotransferase; HDL-C: High-density lipoprotein cholesterol; non-HDL-C: Non-high-density lipoprotein cholesterol; LDL-C: Low-density lipoprotein cholesterol; eGFR: Estimated glomerular filtration rate; hs-cTnT: High-sensitivity troponin T; NT-proBNP: N-terminal pro-B-type natriuretic peptide.

### Comparison of RDW among different study groups

**Fig 3:**
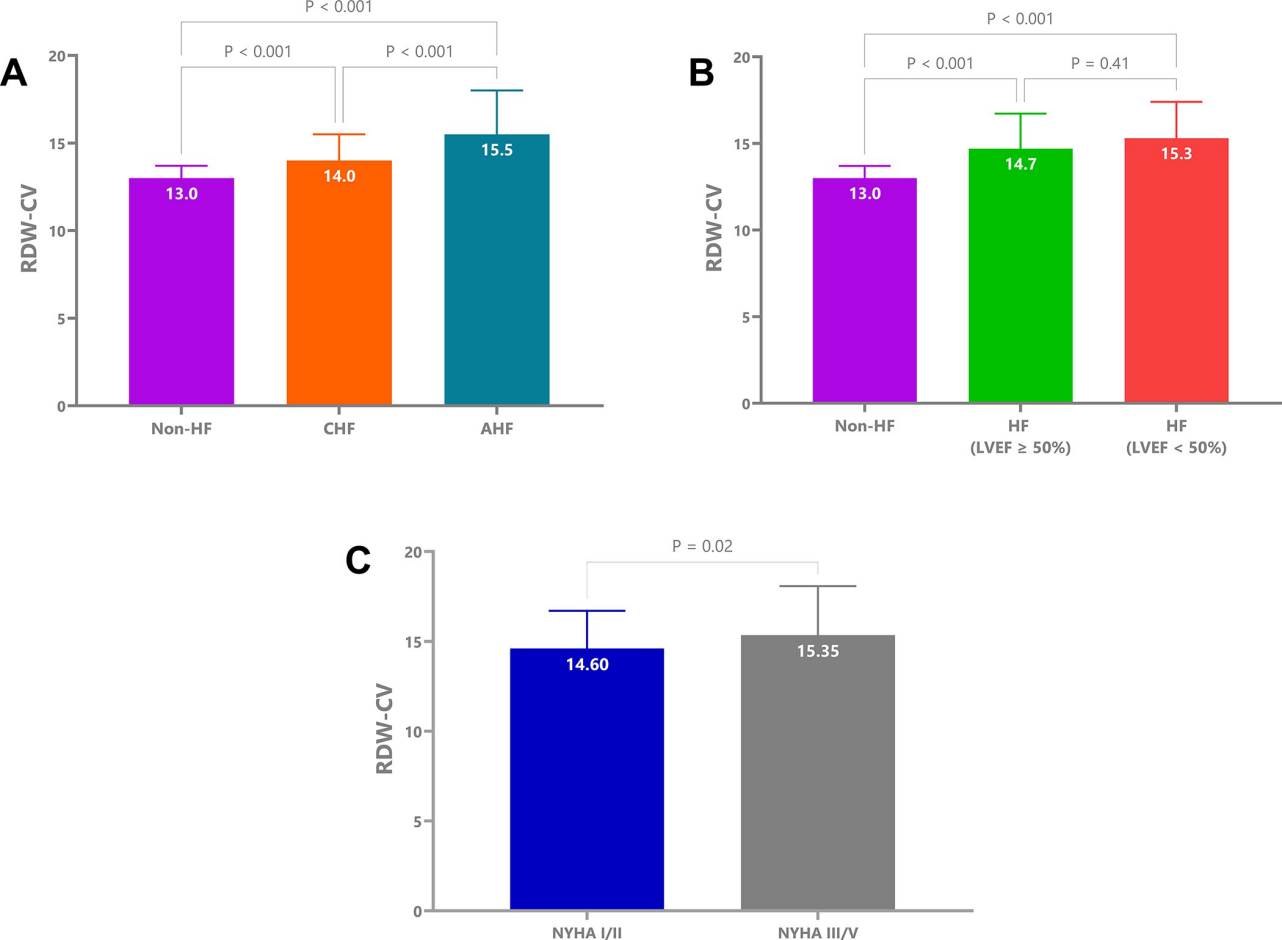
Comparison of RDW-CV among different study groups. (A) Comparison of RDW-CV among the non-HF, CHF, and AHF groups. (B) Comparison of RDW-CV in HF patients with LVEF ≥ 50% and < 50%. (C) Comparison of RDW-CV between the NYHA I/II and NYHA III/IV groups. RDW-CV: Red blood cell distribution width coefficient of variation; HF: Heart failure; CHF: Chronic heart failure; AHF: Acute heart failure; LVEF: Left ventricular ejection fraction; NYHA: New York Heart Association.

We observed significantly higher RDW-CV levels in AHF patients compared to the non-HF group. Additionally, HF patients with preserved LVEF had lower RDW-CV values than those with LVEF < 50%. Moreover, RDW levels increased with higher NYHA classifications. Detailed findings are illustrated in **Fig 3**.

### The value of RDW for the detection of AHF

**Fig 4:**
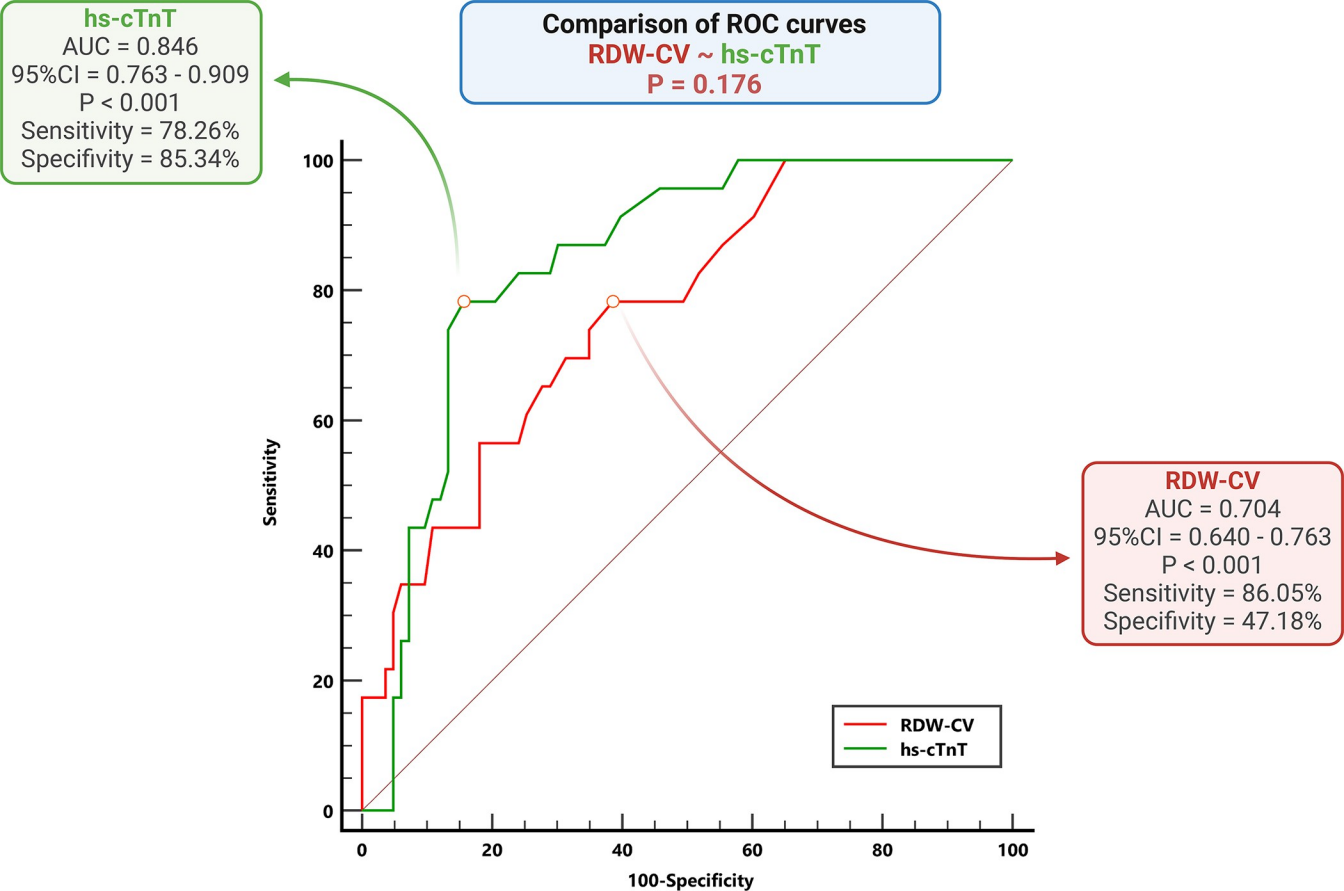
Receiver operating characteristic curves for detecting AHF among HF patients. Comparisons of the differences between the AUCs of RDW-CV and hs-cTnT using the Hanley & McNeil method. ROC: Receiver operating characteristic; AUC: Area under the curve; RDW-CV: Red blood cell distribution width coefficient of variation; hs-cTnT: High-sensitivity troponin T; AHF: Acute heart failure.

The value of RDW-CV for detecting AHF in patients with HF was elucidated through receiver operating characteristic (ROC) analysis, which yielded an AUC of 0.704 (p < 0.001). The identified cutoff point was 13.85%, with a sensitivity of 86.05% and a specificity of 47.18%. Comparative analysis between RDW-CV and hs-cTnT revealed a superior AUC for the latter. Nevertheless, we found the discrepancy nonsignificant by employing the Hanley and McNeil methods to discern the statistical significance between these AUCs (p > 0.05). **Fig 4** further illustrates the diagnostic utility of AHF across various indices, including RDW-CV and hs-cTnT.

### Correlation analysis of RDW and clinical and laboratory indices in HF patients

**Fig 5:**
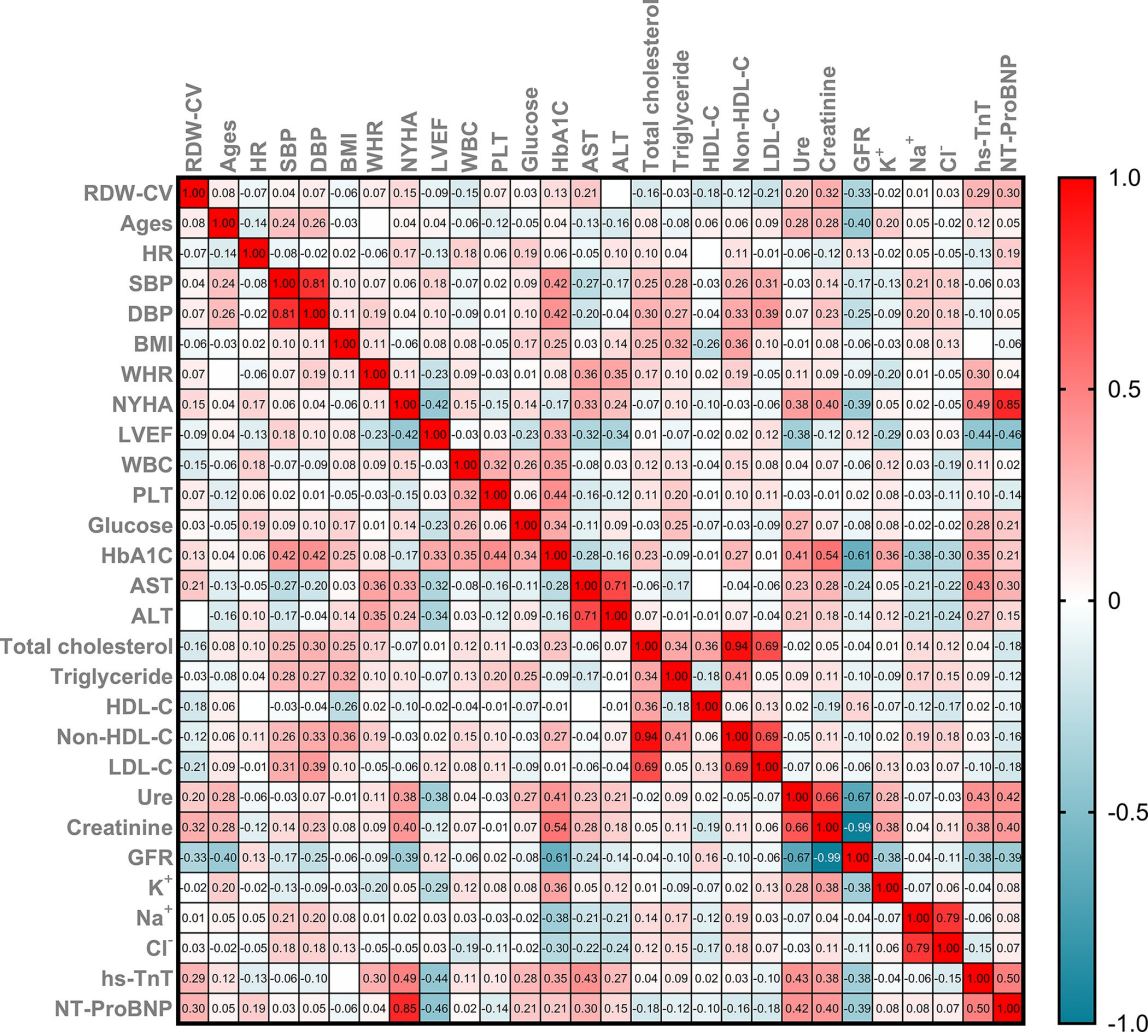
The heatmap shows the correlation of RDW-CV with clinical and subclinical indices in HF patients. HR: Heart rate; SBP: Systolic blood pressure; DBP: Diastolic blood pressure; BMI: Body mass index; WHR: Waist hip ratio; NYHA: New York Heart Association; LVEF: Left ventricular ejection fraction; WBC: White blood cell; RDW-CV: Red blood cell distribution width coefficient of variation; PLT: Platelet; MPV: Mean platelet volume; HbA1c: Hemoglobin A1c; AST: Aspartate aminotransferase; ALT: Alanine aminotransferase; HDL-C: High-density lipoprotein cholesterol; non-HDL-C: Non-high-density lipoprotein cholesterol; LDL-C: Low-density lipoprotein cholesterol; eGFR: Estimated glomerular filtration rate; hs-cTnT: High-sensitivity troponin T; NT-proBNP: N-terminal pro-B-type natriuretic peptide.

The RDW-CV index correlated positively with NT-proBNP and hs-cTnT. Conversely, the RDW-CV index correlated negatively with the eGFR. Furthermore, the heatmap clearly depicts other parameters (**Fig 5**).

### Logistic regression analysis

**Fig 6:**
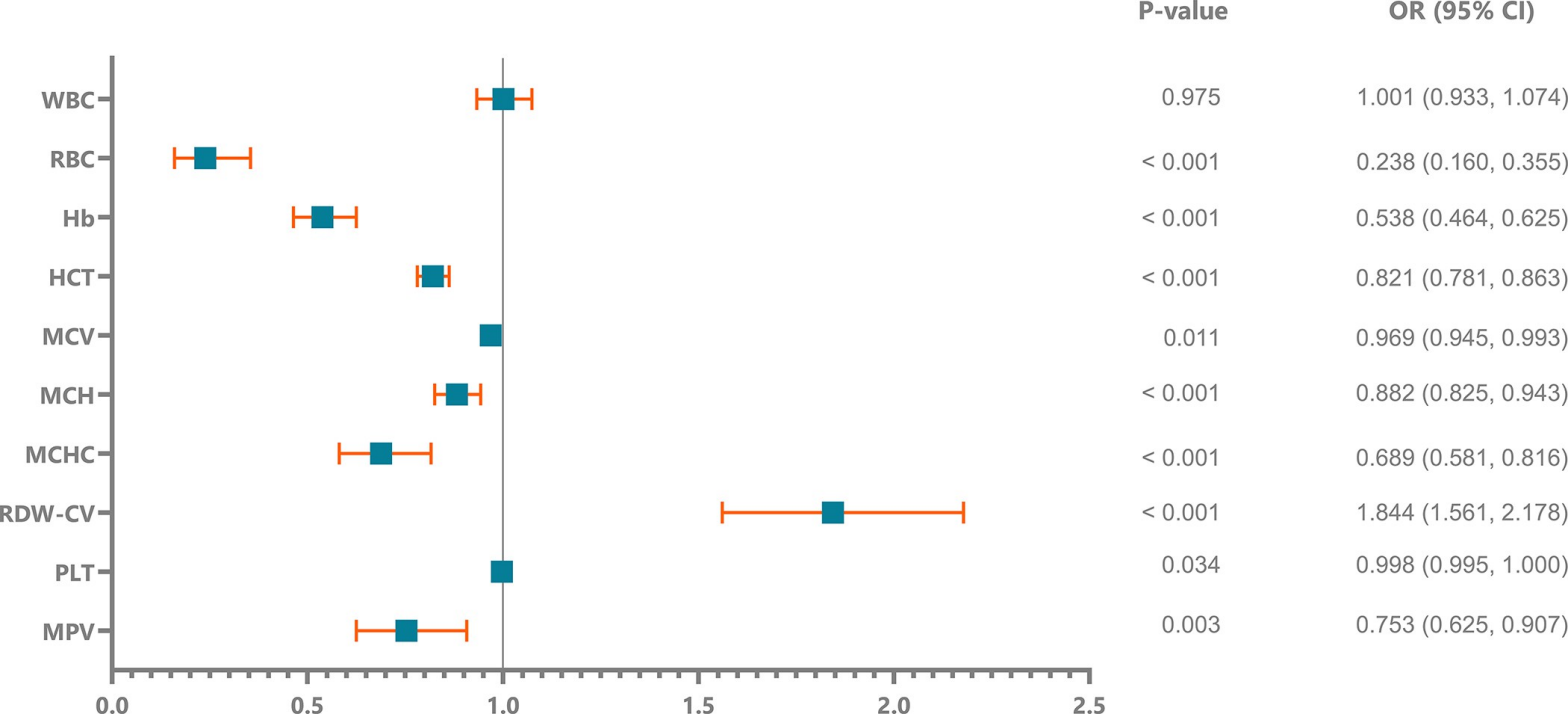
HF susceptibility was predicted via logistic regression using hematologic parameters as covariates. According to the univariate analysis, elevated RDW-CV and lower RBC and Hb levels were identified as risk factors for HF. WBC: White blood cell; RBC: Red blood cell; Hb: Hemoglobin; HCT: Hematocrit; MCV: Mean corpuscular volume; MCH: Mean corpuscular hemoglobin; MCHC: Mean corpuscular hemoglobin concentration; RDW-CV: Red blood cell distribution width coefficient of variation; PLT: Platelet; MPV: Mean platelet volume.

**Fig 6** portrays the outcomes of logistic regression analyses for RDW-CV in HF patients, underscoring elevated RDW-CV levels as significant risk factors for HF. Additionally, **Table 3** outlines independent risk factors identified in the complete blood count of AHF patients, where RDW-CV > 13.85% were recognized as independent risk factors with odds ratios of 2.644 (95% CI, 1.190–5.875; p = 0.017), respectively.

**Table 3:** Logistic regression analysis for prediction of AHF in HF patients.

Factors	Univariable				Multivariable			
	OR ratio	p value	95% CI		OR ratio	p value	95% CI	
**WBC (K/μL)**	1.102	0.103	0.981	1.237				
**RBC (M/μL)**	0.458	**< 0.001**	0.304	0.689	0.313	**0.005**	0.140	0.701
**Hb (g/dL)**	0.737	**< 0.001**	0.643	0.846	0.085	0.074	0.006	1.270
**HCT (%)**	0.915	**< 0.001**	0.874	0.957	2.294	0.068	0.940	5.595
**MCV (fL)**	0.998	0.877	0.971	1.026				
**MCH (pg)**	0.966	0.339	0.899	1.037				
**MCHC (g/dL)**	0.760	**0.009**	0.619	0.934	1.397	0.385	0.657	2.967
**PLT (K/μL)**	0.998	0.299	0.995	1.001				
**MPV (fL)**	1.145	0.271	0.899	1.458				
**RDW-CV > 13.85%**	3.612	**< 0.001**	1.755	7.432	2.644	**0.017**	1.190	5.875

Bold values indicate statistical significance at the p < 0.05 level. CI: Confidence interval; OR: Odds ratio; WBC: White blood cell; RBC: Red blood cell; Hb: Hemoglobin; HCT: Hematocrit; MCV: Mean corpuscular volume; MCH: Mean corpuscular hemoglobin; MCHC: Mean corpuscular hemoglobin concentration; RDW-CV: Red blood cell distribution width coefficient of variation; PLT: Platelet; MPV: Mean platelet volume.

## Discussion

RDW is a simple, fast, cost-effective hematological parameter with results routinely provided in complete blood count. Our study observed that RDW levels were significantly higher in the HF group compared to the healthy control group. Additionally, RDW tended to increase further within the HF group in AHF cases. Furthermore, the study also identified increased RDW as an independent factor for AHF. Therefore, RDW proved to be a valuable prognostic marker for AHF in patients with HF.

### RDW in HF patients

In our study, RDW in HF patients tended to be greater than that in non-HF patients (p < 0.001) (**Fig 3**). Moreover, patients with elevated RDW were more likely to have HF than those with normal RDW **(Fig 6**). Celik et al. (2012) also reported that the HF group had greater RDW than the control group [22]. Additionally, Yuxiang Dai et al. (2014) revealed that half of HF patients had an RDW above the upper limit of the normal range [23].

In our study, AHF patients had greater RDW than CHF patients and controls (p < 0.001). Additionally, NYHA III/IV patients had significantly greater RDW than NYHA I/II patients (p = 0.02) (**Fig 3**). Similarly, Jaewon Oh et al. (2009) reported increased RDW in AHF patients [24].

Currently, the pathophysiological mechanism underlying the increased RDW in HF patients remains incompletely understood. Some recent literature suggests that inflammation, activation of the neurohumoral system, and adrenergic activation in HF patients may influence the erythrocyte maturation process, leading to elevated RDW [15,25,26]. RBC is formed in the bone marrow from erythroid colony-forming unit-erythroid progenitors and undergoes maturation into mature erythrocytes through a series of developmental stages [27]. Disruption of various biological pathways, such as those involved in aging, inflammation, oxidative stress, nutritional deficiencies, impaired renal function, dyslipidemia, and alterations in RBC deformability or fragmentation, has been linked to impaired erythropoiesis, resulting in increased RDW [15,25,28–30].

Inflammation is recognized as a significant contributor to the pathophysiology of HF [31]. In HF, both cell- and cytokine-mediated inflammatory pathways are activated, causing bone marrow dysfunction and the premature release of erythrocytes into the bloodstream. Inflammation inhibits bone marrow function and iron metabolism, while proinflammatory cytokines inhibit erythropoietin-induced erythrocyte maturation and proliferation, thus increasing the RDW [32]. A correlation between RDW and inflammatory markers such as interleukin-6 (IL-6) and C-reactive protein (CRP) has been reported [15]. Proinflammatory cytokines such as tumor necrosis factor-α (TNF-α), IL-1β, and IL-6 have been shown to reduce renal erythropoietin (EPO) synthesis, desensitize erythroid progenitor cells to EPO and inhibit erythropoietin receptor expression, resulting in impaired erythroid progenitor cell proliferation, reduced RBC maturation, and increased RDW [33].

Neurohormonal activation is recognized as one of the primary mechanisms driving the progression of HF, and therapeutic inhibition of neurohormonal systems has emerged as the fundamental approach in modern pharmacotherapy for HF [34]. Erythrocyte progenitor cells are stimulated by the activation of neurohumoral and adrenergic systems, leading to reduced erythropoiesis and, consequently, elevated RDW. Thus, regardless of the underlying cause, this activation directly contributes to the increase in RDW [22,35].

HF often coexists with several comorbidities, among which renal dysfunction is significant. Cardiac and renal diseases intricately interact in both acute and chronic settings, forming a complex bidirectional relationship. Pathophysiologically, these conditions share common pathways, including inflammatory and direct cellular immune-mediated mechanisms, stress-mediated and neurohormonal responses, metabolic and nutritional alterations such as bone and mineral disorders, changes in hemodynamics and acid-base or fluid status, and the onset of anemia [36]. These mechanisms contribute to renal impairment in HF patients. Additionally, these mechanisms have also been implicated in increasing RDW. Our study revealed an inverse correlation between RDW and eGFR, with r_s_ = -0.33, as depicted in **Fig 5**.

### The ability of RDW to detect AHF in HF patients

Previous studies have shown that an increase in RDW is associated with an increased risk of hospitalization due to AHF [17,37]. Ferreira et al. (2013) demonstrated that a high RDW at admission predicts a slower diuretic response [38]. These findings provide us with positive insight into RDW levels in AHF patients.

In our study, RDW demonstrated an ability to detect AHF in HF patients, with an AUC of 0.704 (p < 0.001). The cutoff value of RDW was 13.85%, with a sensitivity of 86.05% and a specificity of 47.18%. Furthermore, the diagnostic value of RDW for AHF was comparable to that of hs-cTnT, with no significant difference in the AUC for detecting acute decompensated HF between these two parameters (**Fig 4**). Hs-cTnT elevation is typical among patients with AHF [39]. An increased troponin level undoubtedly predicts outcomes in patients with AHF; the more significant the elevation, the poorer the prognosis. It is crucial to recognize that cardiac troponin indicates myocardial necrosis and is mainly employed for acute coronary syndrome diagnosis, which may precipitate an AHF episode. In this context, alterations in troponin levels over successive tests and the highest recorded level offer significant diagnostic and prognostic insights [40,41]. We found that an inexpensive and widely available test, such as RDW, has an equivalent value to that of hs-cTnT in detecting AHF.

Additionally, in our study, NT-proBNP exhibited a positive correlation with RDW in HF patients (**Fig 5**). The value of NT-proBNP in diagnosing and ruling out AHF has not been disputed. Numerous studies have demonstrated the excellent role of NT-proBNP in HF patients, especially in AHF patients [42–45]. However, in developing countries, the cost of performing NT-proBNP tests is prohibitively high, and these tests are not available at primary healthcare facilities. Conversely, RDW is readily available and very inexpensive. Our study indicates that the positive correlation between RDW and NT-proBNP could open the door for applying this index in primary healthcare facilities and developing countries such as Vietnam.

### Clinical applications and future directions

In clinical practice, RDW is a straightforward parameter readily available in complete blood count analysis. Complete blood count analysis, an inexpensive routine test, is widely employed for diagnosing various pathologies, particularly cardiovascular diseases [46,47]. Analyzing complete blood count yields multiple applications in clinical practice, especially within the cardiology specialty [48]. Leveraging the availability of RDW without incurring additional costs for further tests when assessing blood counts is advantageous in cardiovascular diseases, including HF [49].

In our study, RDW has demonstrated significant value in diagnosing AHF. However, a deeper analysis reveals that while RDW exhibits high sensitivity, its specificity is relatively limited. Routine RDW detection can be implemented in nearly all clinical laboratories. This observation indicates that RDW can serve as a valuable screening tool, aiding clinicians in the early detection of AHF severity and providing a basis for further specialized testing. However, combining RDW with other clinical and laboratory tools is necessary for a more accurate diagnosis, rather than using RDW as a standalone diagnostic method.

In addition to RDW, novel biomarkers such as platelet distribution width, MPV, and the neutrophil-to-lymphocyte ratio derived from blood count analysis offer simple methods to assess cardiovascular disease status [48,50]. Given their potential, conducting specific studies to comprehensively evaluate the value of these indices, along with RDW, in clinical practice is essential for the future.

### Strengths and limitations

Strengths of this study include detailed data on clinical and laboratory parameters. Our study is the first of its kind conducted on the population of Vietnam, particularly in Central Vietnam, which could provide evidence to support further related research and assist clinical practitioners with an additional, readily available tool to evaluate AHF patients. However, our study has several limitations that warrant consideration. First, relying solely on the RDW without concurrent inflammatory markers such as C-reactive protein and gamma-glutamyl transferase may result in an incomplete assessment of inflammatory status, given the susceptibility of the RDW to various conditions. Additionally, we lacked control over laboratory parameters linked to RDW variation and utilized a single blood analyzer machine (Sysmex XS-1000i, Japan) without intermachine comparisons. Despite efforts to mitigate selection bias through sequential sampling during routine HF visits, the initial conduct of our study at a single hospital with a cross-sectional approach may limit its generalizability. Moreover, the exclusivity of our study cohort to HF patients in central Vietnam further restricts generalization. Acknowledging the lack of serum iron ion data, an essential factor that could affect our results, future investigations will address this issue and consider the impact of unmeasured variables such as iron deficiency to strengthen the study’s reliability.

## Conclusions

In the HF cohort, RDW was significantly greater than that in the non-HF cohort, and RDW was positively correlated with NT-proBNP concentrations. Additionally, RDW proved to be a valuable marker for detecting AHF in HF patients. This study underscores the importance of further RDW research in HF to enhance understanding of its pathophysiology and improve risk assessment in HF management. Given its affordability and accessibility, the RDW offers potential for application in resource-constrained environments.

## Supporting information

S1 Text (DOCX)
