# Supplementary material for: Assessing red blood cell distribution width in Vietnamese heart failure patients: A cross-sectional study
Source: PLoS One. 2024 Jul 23;19(7):e0301319. doi: 10.1371/journal.pone.0301319 (PMC11265657; doi:10.1371/journal.pone.0301319)
Supplement: S1 Text — (DOCX) [file pone.0301319.s001.docx]

**Abbreviations:** ACEi/ARB, angiotensin-converting enzyme inhibitor/angiotensin receptor blocker; AHF, acute heart failure; ALT, alanine aminotransferase; ARNI, angiotensin receptor neprilysin inhibitor; AST, aspartate aminotransferase; AUC, area under the curve; BMI, body mass index; CABG, coronary artery bypass graft; CHF, chronic heart failure; CI, confidence interval; CKD, chronic kidney disease; CRP, C-reactive protein; CV, coefficient of variation; DBP, diastolic blood pressure; eGFR, estimated glomerular filtration rate; EPO, erythropoietin; Hb, hemoglobin; HbA1c, hemoglobin A1c; HCT, hematocrit; HDL-C, high-density lipoprotein cholesterol; HF, heart failure; HR, heart rate; hs-cTnT, high-sensitivity troponin T; IL-6, interleukin-6; LDL-C, low-density lipoprotein cholesterol; LVEF, left ventricular ejection fraction; MCH, mean corpuscular hemoglobin; MCHC, mean corpuscular hemoglobin concentration; MCV, mean corpuscular volume; MPV, mean platelet volume; MRA, mineralocorticoid receptor antagonist; non-HDL-C, non-high-density lipoprotein cholesterol; NT-proBNP, N-terminal pro-B-type natriuretic peptide; NYHA, New York Heart Association; OR, odds ratio; PLT, platelet; RBC, red blood cell; RDW, red cell distribution width; RDW-CV, red blood cell distribution width coefficient of variation; ROC, receiver operating characteristic; SBP, systolic blood pressure; SD, standard deviation; SGLT2i, sodium-glucose cotransporter 2 inhibitor; TNF-α, tumor necrosis factor-α; WBC, white blood cell; WHR, waist–hip ratio.
